# Intra- and Inter-observer Variability in Different Methods of Measuring Carpal Collapse

**DOI:** 10.5704/MOJ.1903.003

**Published:** 2019-03

**Authors:** S Agrawal, T Chabra, S Pandey, P Bhardwaj

**Affiliations:** Department of Orthopaedics, Grande International Hospital, Kathmandu, Nepal; * Department of Orthopaedics, Ganga Hospital, Coimbatore, India; ** Department of Plastic Surgery, Welwitschia Hospital, Walvis Bay, Namibia; *** Department of Hand Surgery, Ganga Hospital, Coimbatore, India

**Keywords:** capitate, carpal height, collapse, ratio, third metacarpal

## Abstract

**Introduction:** Carpal collapse of wrist occurs in disorders like rheumatoid arthritis and Kienbock's disease. Three techniques have been described to measure carpal collapse. First, the carpal height ratio (CHR), measured by dividing carpal height by 3rd metacarpal length. Second, the revised carpal height ratio (RCH ratio), measured by dividing carpal height by length of capitate. Third, capitate radius distance (CR index), measured by shortest distance between distal edge of radius and the proximal edge of capitate. The index publications describe good reliability of all these but which method out of the three is best in terms of intra- and inter-observer variability is not known. The purpose of this study was to find out which method had the least inter- and intra-observer variability for determining carpal collapse.

**Materials and Methods:** Fifty normal wrist postero-anterior radiographs were studied by three assessors who measured CHR, RCH ratio and CR index separately. The measurements were repeated after one month by all the three observers. The results were then statistically analysed.

**Results:** The p-value was <0.001 in all the three assessors in CR index meaning that the intra-observer variability was least in CR index. For the inter-observer variability intra class coefficient of 0.9 indicated that the CR index has the least variability.

**Conclusion:** CR index is the most reproducible method to measure carpal collapse. The method which provides accurate measurement of carpal collapse will allow better staging of carpal disorders.

## Introduction

**Fig. 1:**
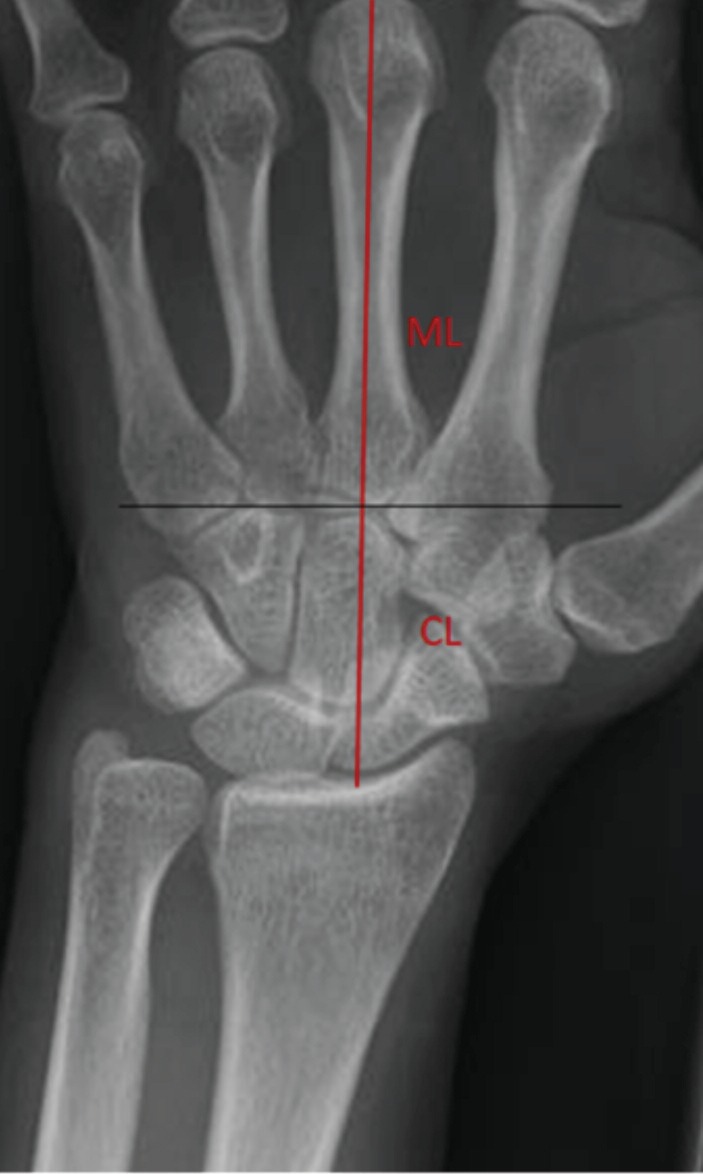
ML is the metacarpal length and CL is the carpal height. Carpal Height Ratio (CHR) = CL/ML.

Carpal collapse occurs in disorders like rheumatoid arthritis, Kienbock’s disease and traumatic injuries to the wrist. It is important to measure the degree of carpal collapse in such disorders^1,2^. The standard accepted method to measure carpal collapse is measuring the carpal height ratio (CHR) which is measured by dividing the carpal height by the length of the 3rd metacarpal with both measurements taken in the long axis of the 3rd metacarpal (Fig. 1)^1,3^. Often it is not possible to measure CHR due to the following two reasons. First, in many wrist radiographs the entire 3rd metacarpal may not be included and second, less commonly the 3rd metacarpo-phalangeal joint may be destroyed by disease process so it is not possible to measure the length of 3rd metacarpal accurately.

**Fig. 2:**
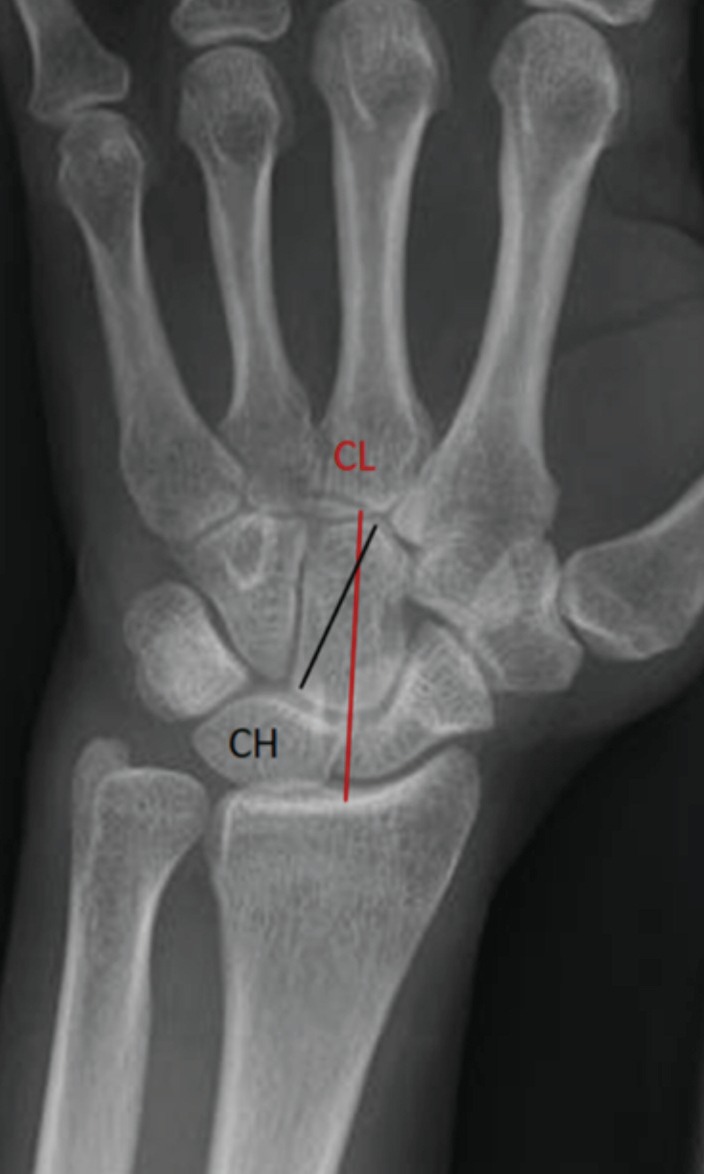
CL is the carpal height and CH is the capitates length. Revised carpal height ratio (RCH ratio) = CL/CH.

A second method, revised carpal height ratio (RCH ratio) has been suggested. This is measured by dividing carpal height by the length of capitate (Fig. 2)^4^. Capitate was referenced because of its well-defined anatomical margins and its rare involvement in disease process. However, a few problems with this method are that the capitate may have different shapes and it differs significantly between men and women.

**Fig. 3:**
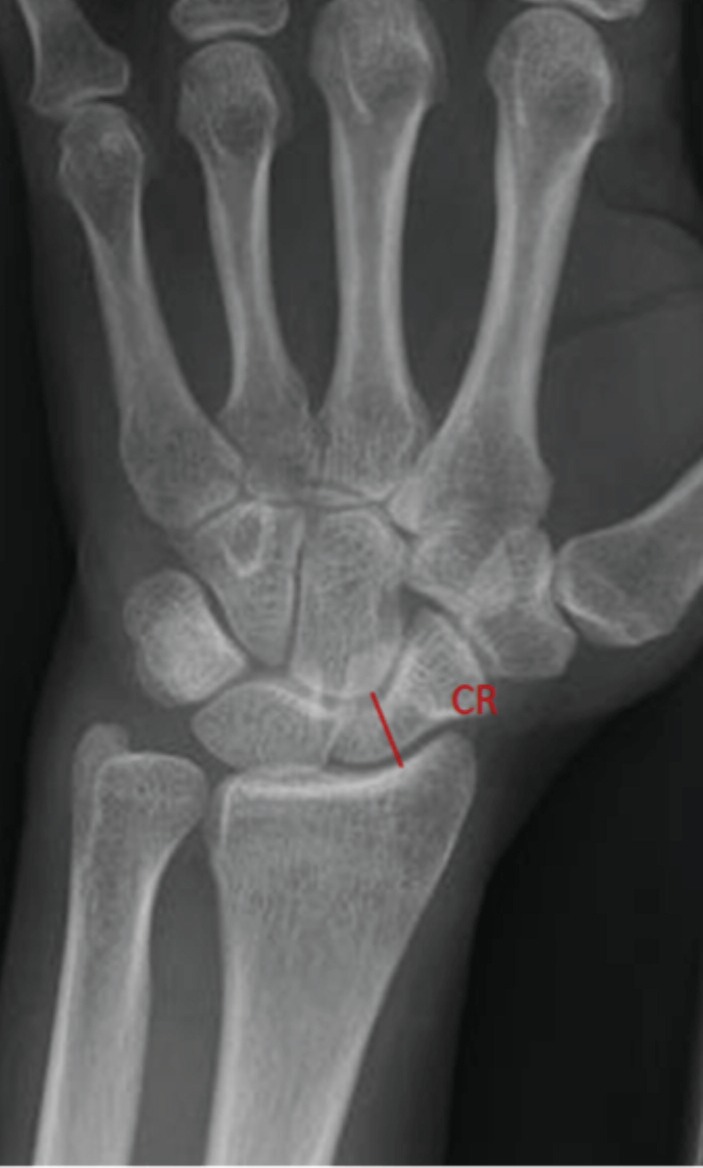
CR Index (CR) is the shortest distance between the distal edge of radius and proximal end of capitates.

Zdravkovic *et al* proposed a third method to indicate carpal collapse, the shortest capitate-radius distance (CR index)^5^ which is measuring the shortest distance between the distal edge of radius and the proximal edge of capitate (Fig. 3).

The index publications of each of these three methods of measuring carpal collapse suggest that their measurement techniques are reliable. However, the question of which is the best among these three methods, as far as inter- and intra-observer reliability is concerned, has yet to be studied. Hence, this study was designed to test the primary aims of these three methods to identify the one with the least variability which could then be employed as a standard for diagnostic evaluation and prognostic outcome of various carpal disorders.

## Materials and Methods

The study was conducted in our hospital. It was a purposive sampling in which fifty normal digital wrist radiographs were selected from the hospital database system. Over a period of three months, a total of 843 radiographs of the wrists had been taken in the hospital and were available in the system database. All the radiographs were screened to eliminate those which had bony injury of any of the metacarpal, carpal, distal radius or ulna bones. Also, those radiographs which had soft tissue shadowing or fat sign suggestive of hematoma or swelling were excluded, leaving 58 wrist radiographs. In eight of these 58 wrist radiographs, the postero-anterior (PA) view was unacceptable and were excluded from the study. The remaining 50 wrist radiographs were deemed appropriate and were included in the study. They were coded and given to the three blinded assessors without any patient details. These assessors were pursuing fellowships in the Hand and Plastic Surgery unit, when the study was conducted. There were three fellows in that academic session of the fellowship. Of the three, two were orthopaedic surgeons and one was a plastic surgeon, with the experience and expertise needed to evaluate the measurement systems.

All the radiographs were independently evaluated by the three assessors and their measurements using the three methods of carpal collapse (CHR, RCH ratio and CR index) which were under evaluation in the study, were recorded and handed over to another independent evaluator. This accounted for the inter-observer data collection component of the study.

CHR was measured by dividing the carpal height by the length of 3rd metacarpal, with both measurements being taken in the long axis of 3rd metacarpal (Fig. 1). RCH ratio was measured by dividing the carpal height, taken in the long axis of 3rd metacarpal, by the longest length of the capitate (Fig. 2). CR index was measured by the shortest distance between the distal edge of the radius and the proximal edge of the capitate (Fig. 3).

The same 50 wrist radiographs were randomly interchanged and given to the same three assessors after a lapse of one month. They repeated the measurements using the three different methods (CHR, RCH ratio and CR index) with their values being assessed by the independent assessor. This accounted for the intra-observer data component of the study.

The data was collected and statistical analysis was done by using SPSS 21 version. Data from all the assessors were described in terms of mean and standard deviation. Intra class correlation coefficient (ICC) was used to describe the intra- and inter-rater reliability and expressed in terms of p-value. A p-value of less than 0.05 was considered to be statistically significant.

## Results

A total of fifty normal wrist postero-anterior radiographs were studied belonging to 36 males and 14 females, in the age range 19 to 78 years. The mean value of measurements obtained by each assessor is shown (Table I). It can be observed from the statistical analysis that there was a strong positive relationship between the two serial measurements among the three observers. This was noted with CHR and CR index, in particular. The CR index (with p-value <0.001 between all three observers) was found to be surpassing the CHR followed by the RCH ratio (Table II). It was also noted that the RCH ratio showed a higher rate of intra-observer variability. Hence, it can be postulated that the CR index has the most reproducible results in terms of intra-observer reliability.

**Table I:** Mean values of measurements by all the observers with standard deviations

	Observer 1		Observer 2		Observer 3	
	1st reading	2nd reading	1st reading	2nd reading	1st reading	2nd reading
CHR	0.50±0.05	0.51±0.03	0.51±0.04	0.50±0.04	0.51±0.04	0.52±0.05
RCH Ratio	1.48±0.18	1.50±0.09	1.57±0.08	1.54±0.08	1.43±0.11	1.47±0.19
CR Index	1.09±0.19	1.17±0.17	1.04±0.15	1.02±0.15	0.96±0.14	1.01±0.15

CHR=carpal height ratio; RCH Ratio=revised carpal height ratio; CR Index=capitate radius distance

**Table II:** Comparison of the first and second readings of individual observers

	Observer 1 ICC (95% CI)	p-Value	Observer 2 ICC (95% CI)	p-Value	Observer 3 ICC (95% CI)	p-Value
CHR 1st reading vs CHR 2nd reading	0.720 (0.50-0.841)	<0.001	0.954 (0.918-0.974)	<0.001	0.610 (0.320-0.777)	0.001
RCH Ratio 1st reading vs RCH Ratio 2nd reading	0.131 (-0.534-0.507)	0.313	0.43 (-0.44-0.183)	0.39	0.501 (0.124-0.716)	0.008
CR Index 1st reading vs CR Index	0.863 (0.470-0.946)	<0.001	0.966 (0.470-0.946)	<0.001	0.887 (0.630-0.952)	<0.001

CHR=carpal height ratio; RCH Ratio=revised carpal height ratio; CR Index=capitate radius distance

The inter-observer variability as studied with intra-class correlation coefficient (ICC) indicated that CHR and CR have an ICC of 0.9 at least in one of the measurements. This in turn showed that both the above have the least inter-observer variability compared to the RCH which never managed to reach the ICC of 0.9 in any of the measurements (Table III).

**Table III:** Inter-observer comparison of the measurements by intra-class correlation coefficient

Measurement ratios	ICC with 95% CI	p-Value (F-test)
CHR I	0.9(0.8-0.9)	<0.01
CHR II	0.7(0.6-0.8)	<0.01
RCH Ratio I	0.2 [(-0.1)-0.5]	0.09
RCH Ratio II	0.5(0.2-0.7)	<0.01
CR Index I	0.9(0.6-0.9)	<0.01
CR Index II	0.8(0.4-0.9)	<0.01

CHR=carpal height ratio; RCH Ratio=revised carpal height ratio; CR Index=capitate radius distance

To summarise, the CR index has the least inter- and intra-observer variability followed by the CHR whose results were not reproducible in intra-observer comparison. RCH ratio was found to have the maximal variability in both inter- and intra-observer comparisons.

## Discussion

Three methods are described in the literature to indicate carpal collapse. They are carpal height ratio (CHR), revised carpal height ratio (RCH) and shortest capitate radius distance (CR index). CHR is the most accepted standard method. However, due to some technical problems like coning down effect of the radiograph beam sometimes the 3rd metacarpal is not visualised fully. Thus, the concept of RCH ratio was suggested by Natrass *et al*^4^. CR index, which subsequently followed, was suggested as an even better indicator of carpal collapse^5^. Hence, we wanted to find out which of the three methods could give a reproducible result when measured by the same observer after a time interval and when two or more observers compared their results.

The normal value for CHR reported is 0.54 ± 0.031,3. The value of CHR in our study (ranging from 0.50 ± 0.04 to 0.51 ± 0.04) is similar to the normal values. Our values do not match with the study done by Schuind *et al* who found the CHR in healthy individuals to range from 0.462 to 0.6086. The normal value for RCH ratio is 1.57 ± 0.05^4^ and in our study, values ranging from 1.43 ± 0.11 to 1.57 ± 0.08 are similar to mentioned normal values. The CR index reported in literature is 0.92^5^ but, in our study, we found that the lowest CR index was 0.69 and highest was 1.59. Nattrass *et al* showed that measurement of RCH ratio was as reliable as that of the CHR with regards to intra- and inter-observer variability^4^. In our study we found that although measurement of CHR was reliable with regards to the intra- and inter-observer variability the same could not be said for that of RCH ratio. The study done by Zdravkovic *et al* showed that measurement of CR index was better in terms of intra- and inter-observer variability when compared to RCH ratio^5^. We also found that the CR index had less variability as compared to RCH ratio in terms of intra- and inter-observer reliability.

We found that the measurement of carpal collapse was most reliable with CR index with regards to intra- and inter-observer variability. The values of CR index vary in normal population. Thus, we suggest that in unilateral disease it is best to take radiographs of both the wrists and compare the diseased side with the normal, both for diagnosis as well as prognosis. Though, in patients with bilateral disease an estimation of carpal collapse on the initial radiographs may not be possible, progression of the disease can be monitored by comparing consecutive measurements of the same wrist. This was suggested by Zdravkovic *et al* and a ratio of new and old CR index of the same wrist which is lower than 0.95 should be considered significant^5^.

One reason why CR index could be more reliable is that only one measurement is utilised, hence reducing the chances of error compared to CHR and RCH ratio which require two measurements. CR index is a reliable indicator in our study because of following two reasons. Firstly, since the measurement points are well defined, the probability of getting same points on repeated examination is more and also it is easy to localize these well-defined points. Secondly, since there is only one shortest distance between the two points (CR index), it is easy to replicate and re-measure.

There are a few limitations of our study. These are purposive sampling technique, small sample size and only normal wrist radiographs being evaluated. It would have been better if a comparison was between normal and affected wrist joints. However, we believe that this study which had a clear question and answer helped us to select the most reproducible of the three measuring techniques for carpal collapse. The comparison with radiographs of diseased and affected wrist can be the subject of a separate study.

## Conclusion

CR index is the most reproducible method for estimating carpal collapse as it has the least inter- and intra-observer variability among all the methods described in the literature.
